# Development of a Flat-Sheet Membrane Gas Exchange Unit for Oxygen Control in Microfluidic Systems

**DOI:** 10.3390/mi17091003

**Published:** 2026-08-25

**Authors:** Anubhav Bussooa, Amaury de Hemptinne, Quentin Galand, Matthieu Briet, Müge Bilgen, Wim de Malsche

**Affiliations:** µFlow Group, Department of Chemical Engineering, Vrije Universiteit Brussel, 1050 Brussels, Belgium

**Keywords:** oxygen, microfluidics, optical oxygen sensor, membrane contactor, COMSOL simulation

## Abstract

Precise control of dissolved oxygen is essential for reproducing physiologically relevant conditions in microfluidic cell culture systems. Here, we present a standalone, polydimethylsiloxane-free gas exchange unit (GEU) which enables controlled oxygenation and deoxygenation of perfused liquids and is suitable for integration with existing microfluidic platforms. The GEU employs a flat-sheet membrane contactor design to achieve efficient gas–liquid mass transfer while remaining independent of the downstream device. Oxygen transfer was experimentally characterised using optical oxygen sensors under different liquid and gas flow conditions. Reoxygenation efficiency decreased with increasing liquid flow rate because of reduced residence time, whereas active airflow through the gas compartment enhanced oxygen transfer. Controlled deoxygenation was achieved by flowing nitrogen through the gas compartment, with higher nitrogen pressures producing progressively lower oxygen levels. Numerical flow simulations demonstrated uniform flow distribution within the device, and a simplified analytical diffusion model accurately predicted the observed oxygen transfer trends. The proposed GEU provides a simple, robust and modular strategy for regulating dissolved oxygen upstream of microfluidic devices without requiring device redesign or specialised fabrication. This approach offers a practical solution for incorporating physiologically relevant oxygen control into a wide range of microfluidic and cell culture applications.

## 1. Introduction

Mammalian cell cultures are most commonly carried out in CO_2_ incubators [1], filled with 95% air and 5% CO_2_, exposing the cells to 19.95% O_2_ [2]. In the human body, the oxygen level is lower than 19.95% O_2,_ and it varies between the different organs and tissues. For example, the oxygen level in arterial blood, kidneys, bone marrow, liver and brain is 13.2%, 9.5%, 6.4%, 5.4% and 4.4%, respectively [2].

The O_2_ level in the cellular microenvironment has a significant effect on cell viability, function, proliferation and differentiation [3,4]. When the oxygen level is above (hyperoxia) or below (hypoxia) the physiological level, potent biological responses are triggered. Hyperoxia dysregulates cellular signalling processes and triggers macromolecular oxidative damage, inducing adverse effects on cell function [5]. Intermittent hypoxia stimulates angiogenesis, while constant hypoxia triggers a stronger, more sustained pro-angiogenic response [4]. Moreover, hypoxia supports the survival and growth of stem and progenitor cells [3]. It is very important that the oxygen level in the in vitro culture systems reflects the in vivo tissue- or organ-specific oxygen level [6] to ensure translatability.

In order to better reproduce in vivo oxygen levels, several research groups have developed microfluidic cell culture devices with integrated oxygen level control [7,8,9,10]. Izadifar et al. [8] developed a dual-channel organ-on-a-chip device using polycarbonate (PC) and polydimethylsiloxane (PDMS). By combining the low oxygen permeability of PC with the high oxygen permeability of PDMS, they generated a semi-isolated cellular microenvironment and achieved oxygen levels in the 2% to 15% range. Kasahara et al. [9] developed a PDMS microfluidic chip enclosed in a 3D-printed micro-incubator for oxygen-controlled microbial cultures. By varying the O_2_ and N_2_ levels in the mini-incubator, they could vary the oxygen level in the culture chamber. Polinkovsky et al. [10] developed a PDMS microfluidic device with a liquid flow layer and a gas flow layer. They applied different ratios of O_2_ and N_2_ into the gas flow layer to generate oxygen levels between 0.0% and 20.9% for culturing *E. coli*. In addition, they demonstrated that injection of CO_2_ into the gas flow layer enabled control of the pH of a buffered solution. Engler et al. [11] used a commercially available hollow fibre cartridge (PermSelect/MedArray/USA) to bring oxygen into a bioreactor system. The cartridge consisted of 3200 thin-walled PDMS hollow fibres, enclosed by a shell. Incubator air was pumped through the fibres while culture medium was flowed on the shell side. Using this system, they were able to maintain stable oxygen levels throughout a 24-h culture period.

In the abovementioned devices, PDMS is used as a gas exchange membrane or bulk material. PDMS has many advantages, such as high gas permeability, high optical clarity and ease of fabrication [12]. However, PDMS suffers from major limitations for cell culture applications. The high permeability of PDMS enables water vapour to easily diffuse out of culture devices, causing changes in osmolality, which adversely affects cells [13]. Cured PDMS contains residual non-crosslinked polymer chains, which leach out into culture medium and incorporate into the cell membrane [14] and adversely affect cellular behaviour [15]. Furthermore, small hydrophobic molecules are readily absorbed by PDMS, significantly disrupting dynamic dosing protocols [16].

Due to the issues with PDMS, some research groups have implemented PDMS-free oxygen control strategies. Sønstevold et al. [17] developed a PC cell culture device, integrating a gas-permeable polymethylpentene film, for culturing epithelial cells at a stabilised oxygen level of 16%. Bunge et al. [18] developed a microfluidic cell culture device using glass, silicon and aluminium. It consisted of an upper chamber for cell culturing, a lower air chamber and an anodised aluminium oxide membrane between them. Using this chip, they were able to maintain > 99% cell viability for 72 h. Buck et al. [19] developed a cell culture device integrating a track-etched nanoporous PC membrane for oxygenation. With this device, they maintained a stable oxygen equilibrium during a 6 h culture period of intestinal epithelial cells. Li et al. [20] developed a micro-tube bioreactor integrating polytetrafluoroethylene (PTFE) tubes for bubble-free oxygenation of microbial cultures. The cultures were carried out under escalating oxygen levels (5% to 20%) and showed an enhanced growth performance. Most of these PDMS-free strategies require specialised engineering equipment for integration of membranes within the cell culture device. However, biology laboratories do not usually have access to such specialised equipment and would benefit from standalone microfluidic gas conditioning units [21], which can be simply connected upstream of cell culture devices.

Flat-sheet membrane contactors have been widely employed to enhance mass transfer between immiscible phases while maintaining physical separation [22,23,24]. These provide a large interfacial area per unit volume and short diffusion distances, resulting in efficient transport processes under laminar flow conditions. Our research group previously implemented such a device for solvent extraction across a membrane interface, and it demonstrated efficient transfer of chemical species between two liquid streams [25]. While originally developed for liquid–liquid extraction, its large membrane surface area, stabilised interface near the membrane and microfluidic geometry also make it suitable for gas–liquid oxygen transfer, requiring only the substitution of one liquid phase with a gas stream. In the present work, this flat-sheet membrane contactor is adapted as a standalone Gas Exchange Unit (GEU) for controlled oxygenation and deoxygenation of perfused liquids. We demonstrate that the GEU enables controllable oxygen regulation by adjusting liquid and gas flow rates, while numerical simulations confirm uniform flow distribution and an analytical diffusion model captures the observed oxygen transfer behaviour. By providing a modular, PDMS-free solution which can be connected upstream of existing microfluidic devices without redesign, the proposed GEU offers a practical strategy for introducing physiologically relevant oxygen control into organ-on-a-chip and other microfluidic cell culture systems.

## 2. Materials and Methods

### 2.1. Materials

**Figure 1:**
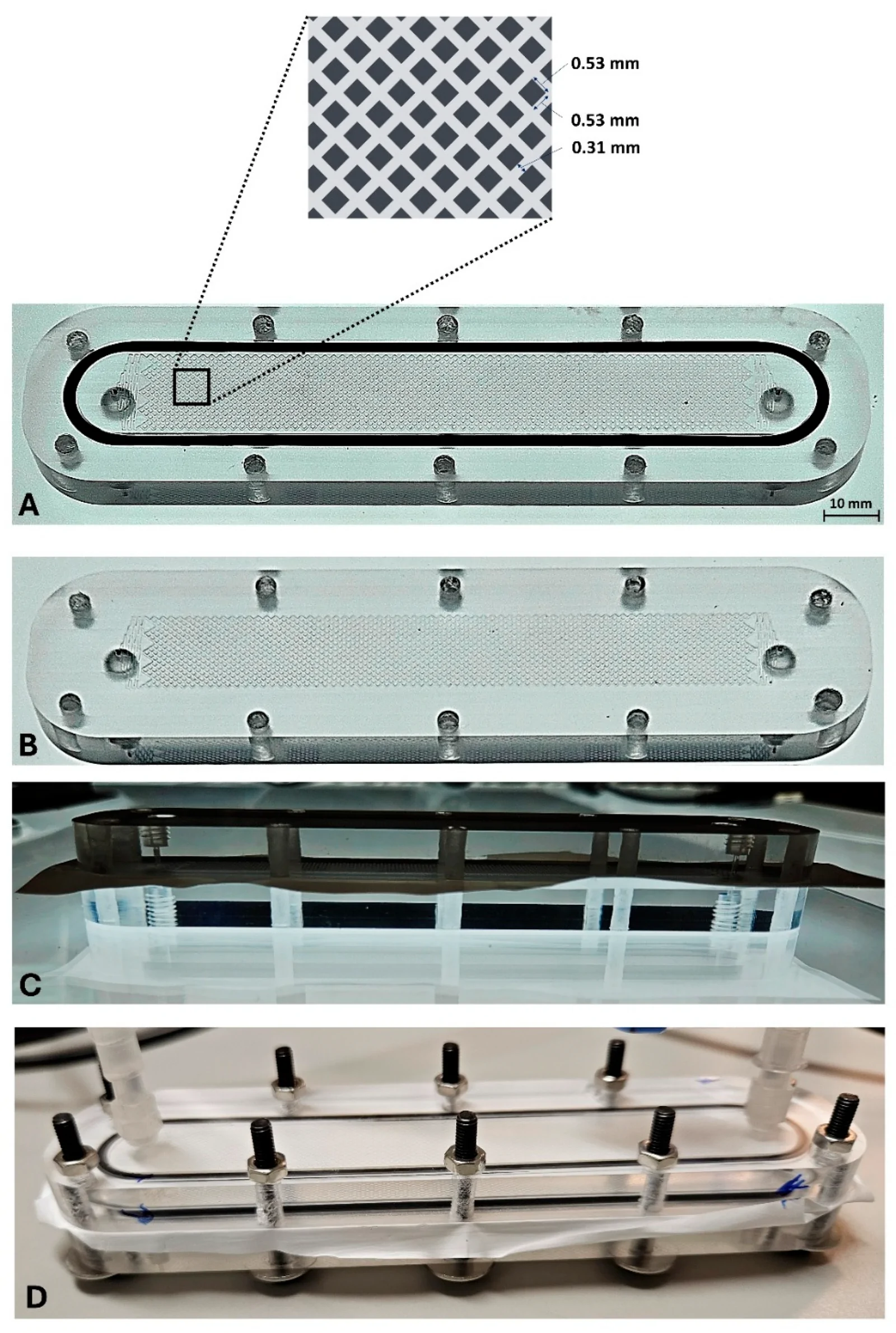
The GEU: top liquid compartment with a zoomed-in section showing 0.53 mm × 0.53 mm micropillars with 0.31 mm separation between micropillars (**A**), the bottom gas compartment with identical design to liquid compartment, except for absence of the O-ring and the groove (**B**), the pre-assembly with the PTFE membrane between the compartments (**C**) and the final assembly with bolts and nuts (**D**).

The GEU was fabricated using 8 mm Polymethyl methacrylate (10611000/PMMA-GS/Eriks/Mol/Belgium) sheets. The 2 compartments (Figure 1A,B) of the GEU were separated using a porous hydrophobic PTFE membrane (T010A304D/Advantec/Tokyo/Japan) as shown in Figure 1C,D. The membrane had a pore size of 0.10 µm, a porosity of 68% and a thickness of 70 µm. Low gas permeability tubing with an internal diameter of 2.29 mm (MF-06460-44/Tygon E-Lab/Saint-Gobain Life Science/Paris/France) was used. The connectors used to connect the tubing to the GEU are shown in Supplementary Figure S3. The liquid inlet tube coming from the flask is connected to a 3/32” Barbed to Female Luer Lock Adapter (P-858/IDEX/Northbrook/IL/USA). This then connects to a Male Slip Luer (10-32 Male UNF) (CIMLSTA-1032M-PC/ISM/Denver/CO/USA), which is then screwed into the 10-32 threaded hole inlet of the GEU. The same connections are used to connect the gas line to the inlet of the gas compartment. At the outlet of the liquid compartment of the GEU, a Male Slip Luer (10-32 Male UNF) is screwed into the 10-32 threaded hole. This then connects to the Flow Through Oxygen Sensor (FTC-PSt3-YOP-4mm/PreSens/Regensburg/Germany).

### 2.2. Design, Fabrication and Operating Principle of GEU

The GEU was designed using SolidWorks 2025 (Dassault Systèmes/Vélizy-Villacoublay/France). A CNC (Computer Numerical Control) milling machine (Datron/Ober-Ramstadt/Germany) was used to micro-mill PMMA sheets. The milling tool used was a 300 µm End mill (Tool ID 0063003E/LMT Tools/Lahr/Germany), with the following parameters: Milling speeds: RPM = 40,000, XY Feed = 100 mm/min and Z Feed = 25 mm/min. The top liquid compartment (Figure 1A) consisted of one inlet, one outlet, a channel with an array of micropillars, 10 through holes and a groove in which a 75.92 mm × 1.78 mm O-ring (11095969/KALREZ/DuPont/Wilmington/DE/USA) was placed. The bottom gas compartment (Figure 1B) consisted of one inlet, one outlet, an array of micropillars (identical to the top liquid compartment) and 10 through holes. The top liquid compartment was pre-assembled onto the bottom gas compartment (micropillars facing each other) with the membrane between (Figure 1C). For the final assembly (Figure 1D), the M3 bolts (560805/RS components/Puurs-Sint-Amands/Belgium) were tightened with a torque of 10 cNm. The membrane and the O-ring created a liquid-tight compartment.

The GEU functions as a flat-sheet membrane contactor in which the liquid and gas streams flow through separate compartments, separated by a hydrophobic porous PTFE membrane. As the membrane pores remain gas-filled, the gas and liquid phases remain physically separated, while oxygen diffuses across the membrane in response to the concentration gradient between the two compartments. The large membrane area and short diffusion distance promote efficient gas–liquid mass transfer, while the extent of oxygen exchange is primarily determined by the liquid residence time within the GEU and the gas composition in the lower compartment.

### 2.3. Deoxygenation Using Flask

**Figure 2:**
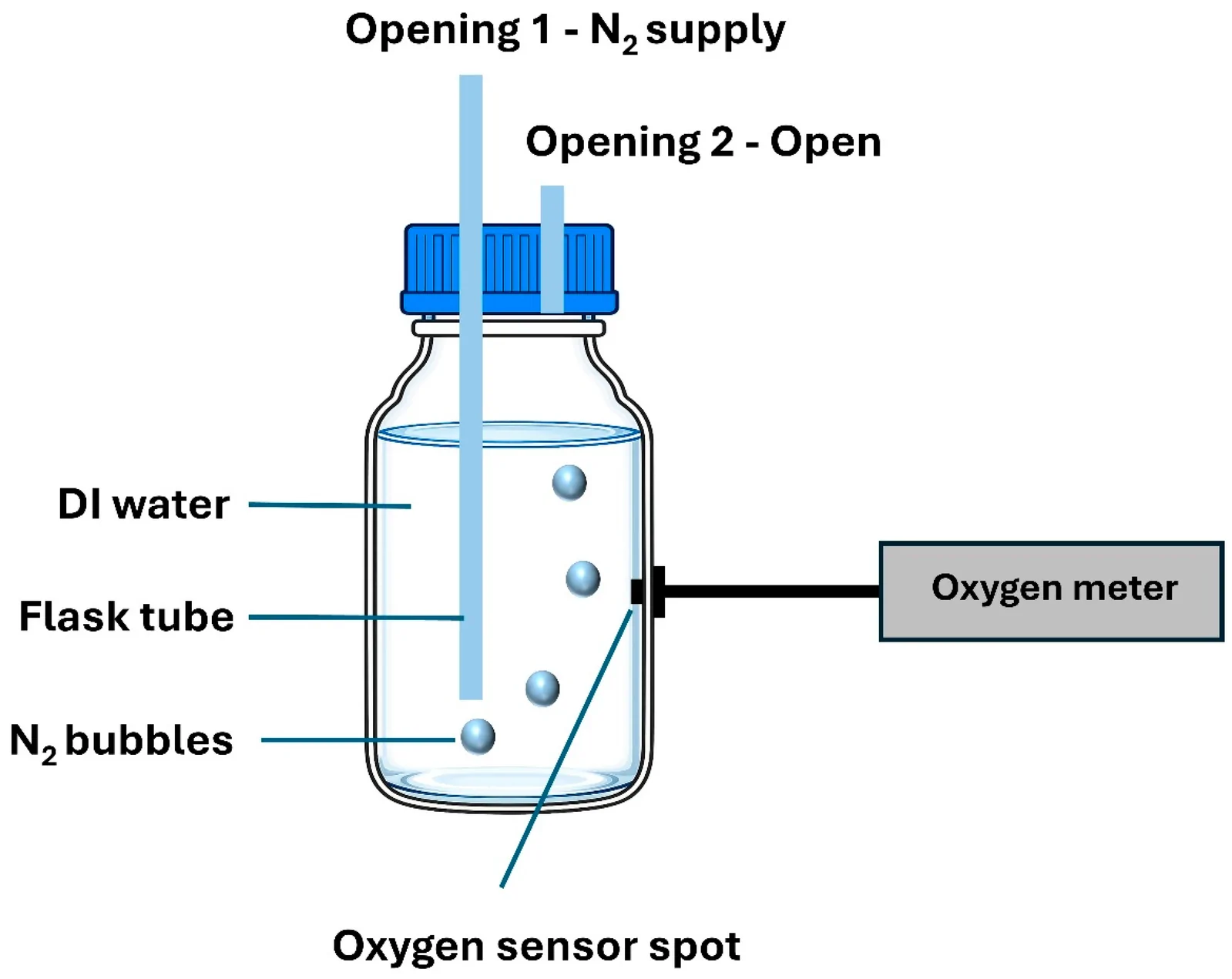
Experimental setup for deoxygenating DI water inside a 200 mL flask.

As depicted in Figure 2, a contactless oxygen sensor spot (SP-PSt3-SA-D5-YOP-US/PreSens/Regensburg/Germany) was glued inside a 200 mL flask (DURAN pressure plus+/DWK Life Sciences/Vineland/NJ/USA). An optical fibre (POF-L2.5-1SMA/PreSens/Regensburg/Germany) was connected to the flask, facing the sensor spot, using a Stick-on Adapter (SOA/PreSens/Regensburg/Germany). The other side of the optical fibre was connected to an oxygen meter (OXY-4 SMA/PreSens/Regensburg/Germany). A temperature probe (TEP-L5-St62-OD5.4/3.3/PreSens/Regensburg/Germany) was also connected to the oxygen meter to monitor the room temperature. The flask was filled with 200 mL of deionised (DI) water and closed using a cap with 2 openings (1 and 2). Opening 1 was used to insert the flask tube close to the bottom of the flask. This flask tube was connected to an N_2_ supply controlled by a pump (Fluigent/Le Kremlin-Bicêtre/France). Opening 2 was left open. The oxygen level of the DI water in the flask was monitored every 10 s using the oxygen meter. From 0 min to 1 min, no N_2_ was bubbled. From 1 min to 15 min, 100 mbar of N_2_ was bubbled.

### 2.4. Reoxygenation Using GEU

**Figure 3:**
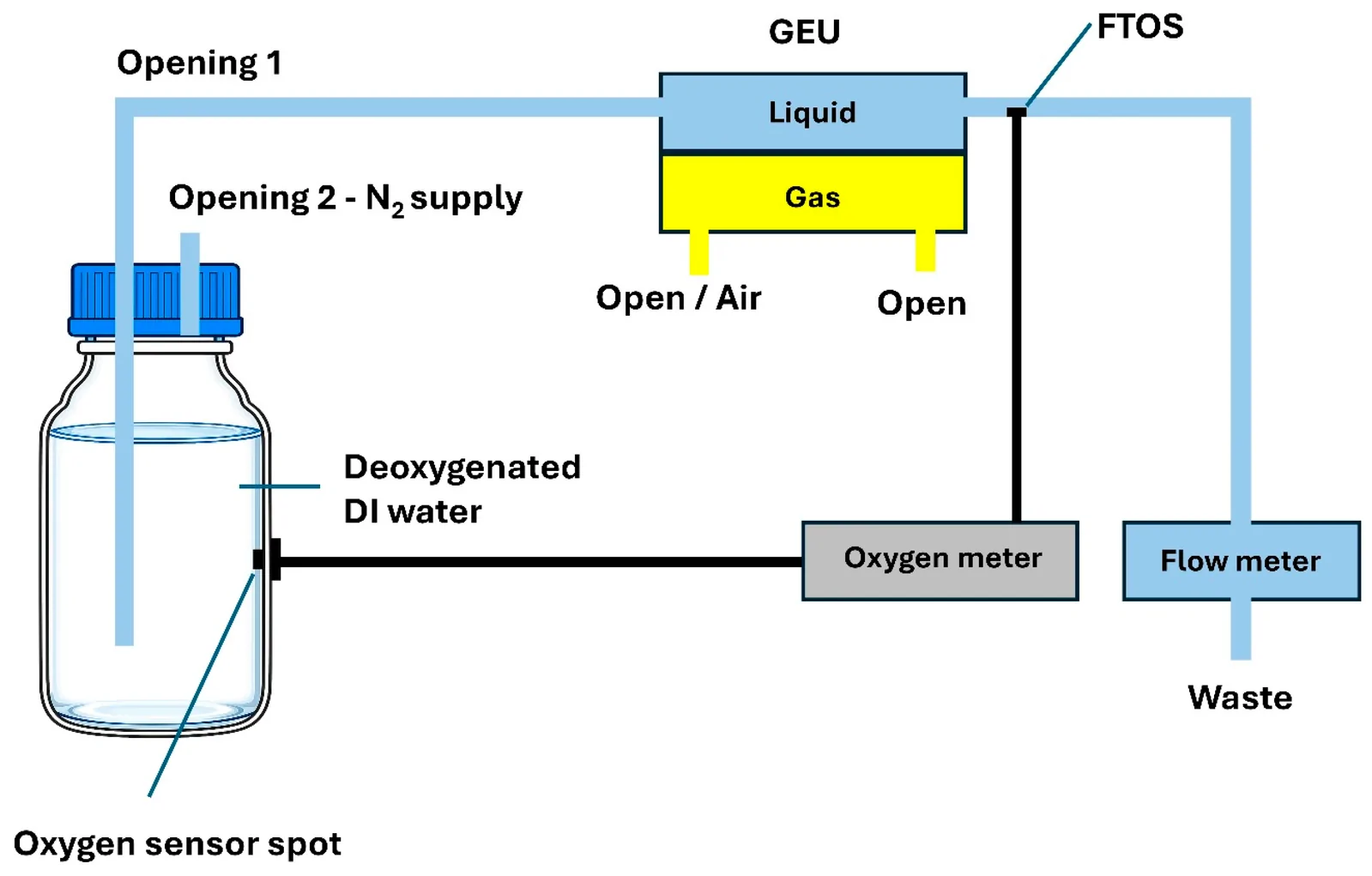
The experimental setup for reoxygenating consists of the flask with integrated oxygen sensor, Gas Exchange Unit (GEU), Flow Through Oxygen sensor (FTOS), flow meter, waste beaker and oxygen meter. For the first series of experiments, the inlet and outlet of the gas compartment of GEU were left open. For the second series of experiments, the inlet of the gas compartment was connected to an air supply, and the outlet was left open.

When the deoxygenation (Section 2.3) was complete (approximately 0% oxygen measured by the sensor spot in the flask), the N_2_ bubbling was stopped. The N_2_ supply was switched from opening 1 to 2, as shown in Figure 3. The flask tube was connected to the GEU, flow-through oxygen sensor (FTC-PSt3-YOP-4mm/PreSens/Regensburg/Germany), flow sensor (Flow Unit/Fluigent/Le Kremlin-Bicêtre/France) and the waste beaker. The flow-through oxygen sensor (FTOS) was connected to the oxygen meter using an optical fibre. The oxygen level was measured every 10 s for a period of 10 min, using the FTOS and the sensor spot inside the flask. In the first series of experiments, the inlet and outlet of the gas compartment of the GEU were left open. From 0 to 2 min, the oxygen measurements were carried out without liquid flow (the liquid flow system was initially filled with air). This acted as the internal control for the experiment. At 2 min, the N_2_ supply (opening 2) was set at 100 mbar. This pushed the deoxygenated DI water through the flask tube into the liquid compartment of the GEU, FTOS, flow meter and waste, successively. The experiment with an applied pressure of 100 mbar was repeated two more times. The experiment was then carried out with applied pressures of 150, 200, 300, 400, 500 and 600 mbar.

In the second series of experiments, a modification was made to the experimental setup. The inlet of the gas compartment was connected to a separate Fluigent pump, and the outlet was left open. This pump was used to flow air through the gas compartment. The airflow and liquid flow were started simultaneously. The air pressure applied to the gas compartment was half that of the liquid compartment. For example:

- When 100 mbar was applied in the liquid compartment, 50 mbar was applied in the gas compartment.
- When 600 mbar was applied in the liquid compartment, 300 mbar was applied in the gas compartment.

### 2.5. Deoxygenation Using GEU

**Figure 4:**
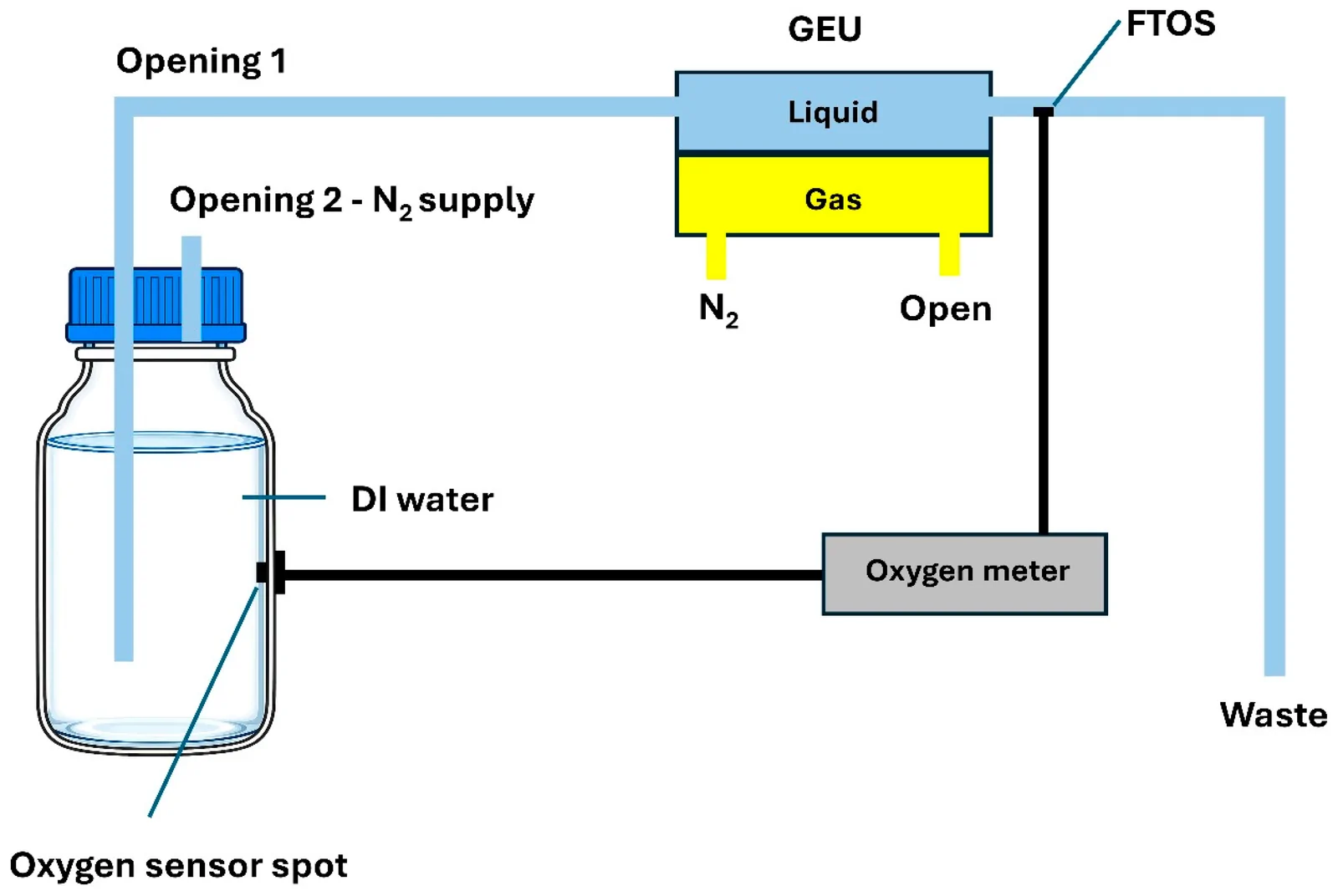
The experimental setup for deoxygenating consists of the flask with integrated oxygen sensor, Gas Exchange Unit (GEU), Flow Through Oxygen sensor (FTOS), waste beaker and oxygen meter. The inlet of the gas compartment of the GEU was connected to a separate nitrogen supply, and the outlet was left open.

In Section 2.3, the DI water was deoxygenated by bubbling N_2_ directly inside the flask. In this section, the DI water was deoxygenated using the GEU. As schematised in Figure 4, the flask was filled with 200 mL of DI water. Opening 2 of the flask was connected to the N_2_ supply. The flask tube was connected to the liquid compartment of the GEU, FTOS and waste. A separate N_2_ supply, controlled by another Fluigent pump, was connected to the inlet of the gas compartment of the GEU, while the outlet was left open. The sensor spot and the FTOS were used to monitor the oxygen level. At 0 min, 200 mbar of N_2_ was applied to opening 2 of the flask to push the DI water through the liquid flow system. From 0 min to 1 min, no N_2_ was applied in the gas compartment. From 1 to 10 min, 10 to 15 min and 15 to 20 min, 2, 3 and 4 mbar N_2_, respectively, were applied.

## 3. Numerical Simulations

**Figure 5:**
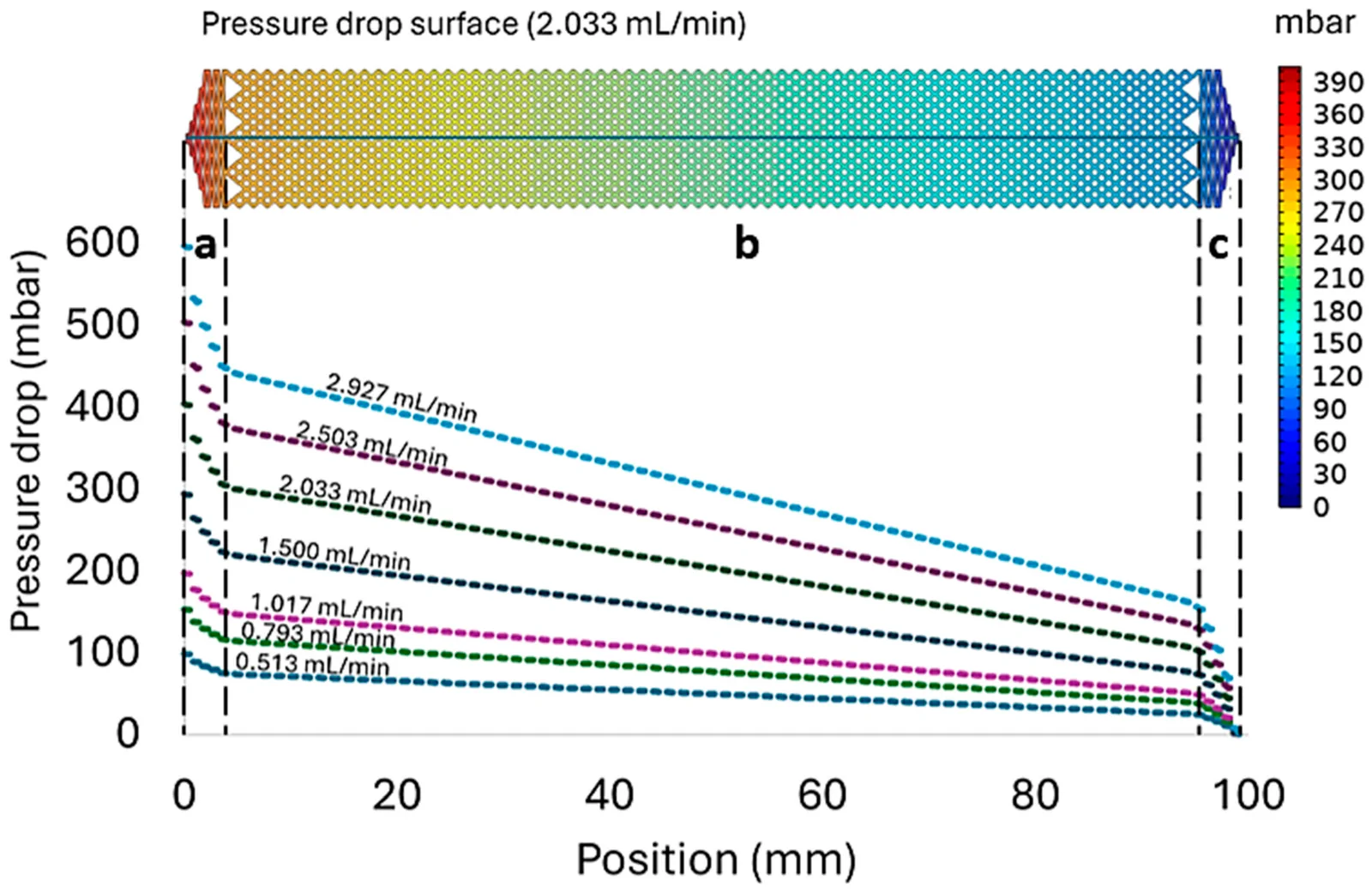
Pressure profile along a longitudinal line (shown as a blue line in the top panel) from inlet to outlet for all investigated flow rates (0.513 to 2.927 mL/min). All curves exhibit a similar shape, characterised by three distinct regions: (**a**) a sharp pressure drop in the inlet distributor, which can be attributed to higher flow velocity resulting from a smaller cross-sectional area, (**b**) a linear decrease across the micropillar-filled chamber and (**c**) a second sharp drop in the outlet distributor, caused by a structure similar to the inlet, reaching zero pressure at the outlet. The linear region indicates a constant pressure gradient, consistent with fully developed flow through a homogeneous structure formed by the micropillars.

Numerical simulations were performed using COMSOL^®^ Multiphysics 5.3a software to characterise the hydrodynamic behaviour of the device. The laminar flow module was employed under stationary conditions, assuming incompressible flow. Boundary conditions were defined by imposing a volumetric flow rate at the inlet and a reference pressure at the outlet. The different flow rates (measured experimentally using a flow meter, corresponding to applied pressures of 100, 150, 200, 300, 400, 500, 600 mbar) were investigated: 0.513, 0.793, 1.017, 1.500, 2.033, 2.503 and 2.927 mL/min, to evaluate the pressure drop and flow distribution within the system. The top panel of Figure 5 shows the pressure distribution within the device for a flow rate of 2.033 mL/min. A gradual decrease in pressure from the inlet to the outlet can be observed. Importantly, the pressure remains uniform across the width of the device, indicating an efficient flow distribution.

**Figure 6:**
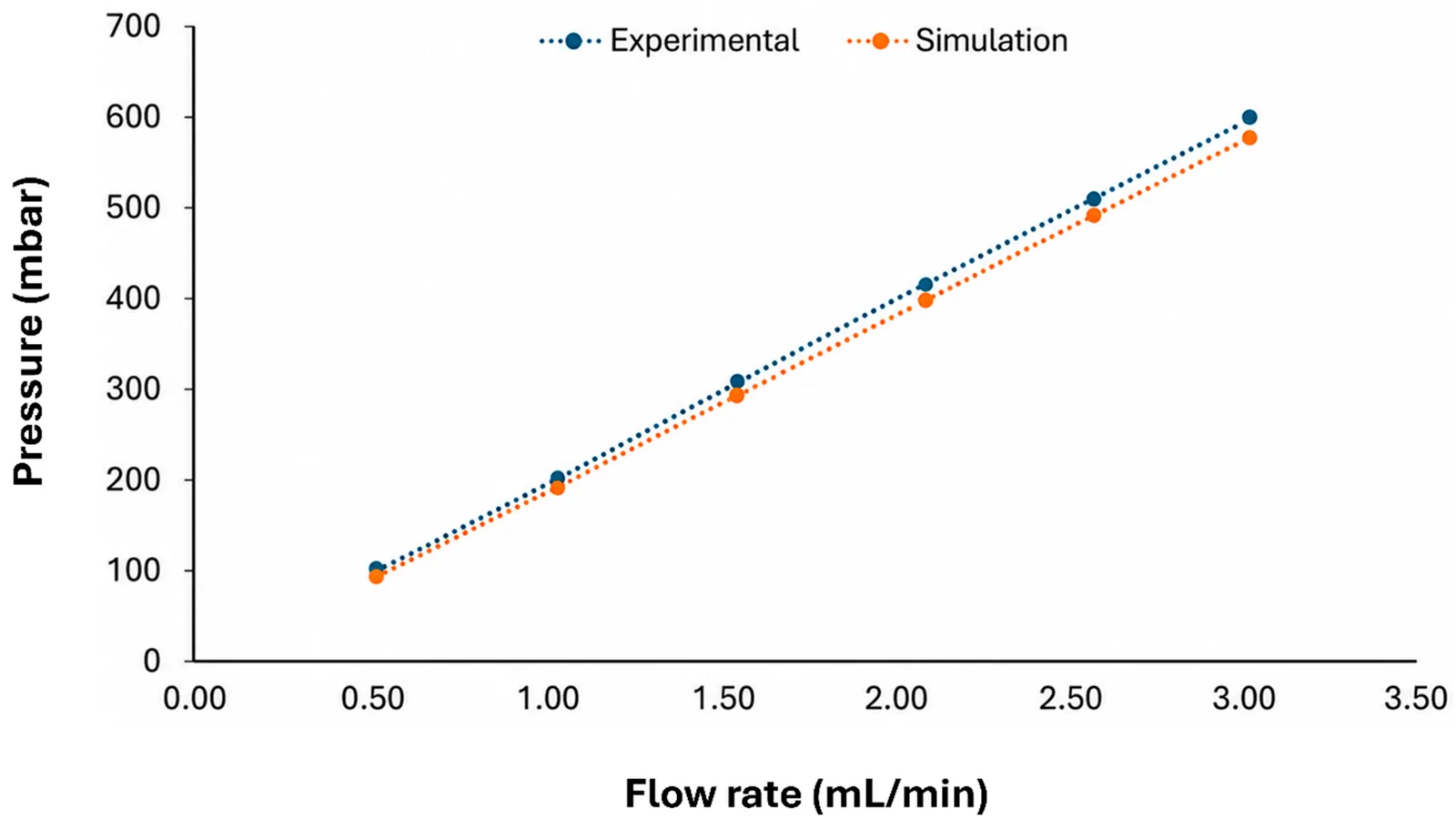
Relationship between inlet pressure drop and flow rate, showing both simulated and experimental data with a strong linear correlation.

The relationship between the pressure drop at the inlet and flow rate is shown in Figure 6, where both simulated and experimental data are reported. The results display a strong linear correlation. Based on this linearity, an extrapolation allows estimation of the maximum achievable flow rate for a given pump pressure (for example, 9.920 mL/min can be achieved with an applied pressure of 2000 mbar), providing a practical guideline for device operation. The pressure drop associated with the inlet and outlet connections was calculated using the Darcy–Weisbach equation [26], for different flow rates. The results indicate that 99.9% of the total pressure drop originates from the GEU. For example, the connection pressure drop is 0.076 mbar at 0.513 mL/min (corresponding to an applied pressure of 100 mbar), while 0.430 mbar was calculated at 2.927 mL/min (corresponding to an applied pressure of 600 mbar).

## 4. Analytical Modelling

**Figure 7:**
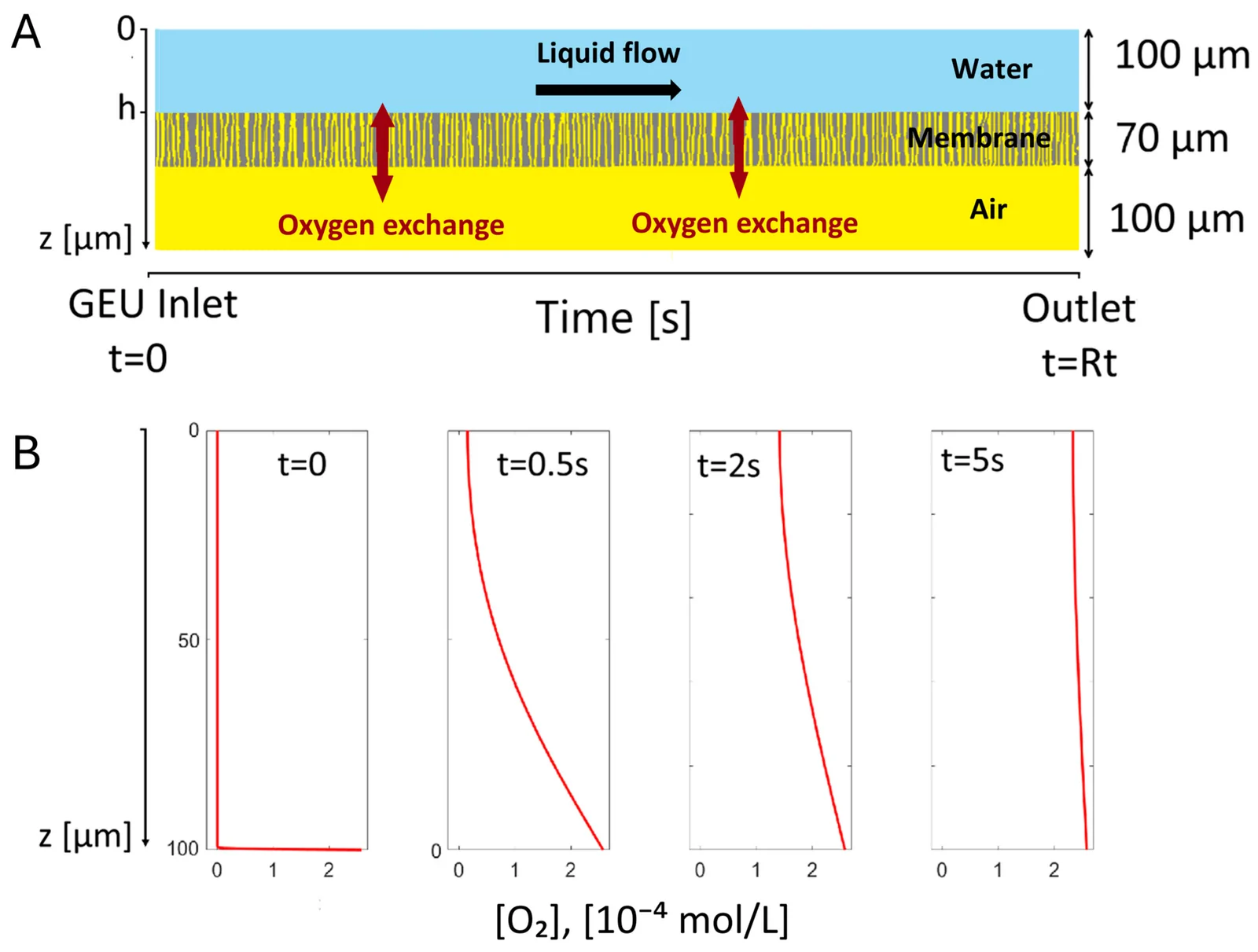
(**A**) Simplified 2D schematic representation of the Gas Exchange Unit. The liquid flows through the GEU in the top compartment, and the bottom compartment is filled with air. The membrane is hydrophobic; the pores of the membrane are filled with air, and the liquid–air interface is located at the h ordinate of the *z*-axis. (**B**) Oxygen transport by diffusion occurs mainly vertically, and the oxygen concentration is from left to right. Oxygen concentration profiles were obtained with the analytical model, considering a perfectly deoxygenated liquid at the inlet of the GEU.

Developing an explicit analytical model of the membrane oxygenator is challenging as this would require simultaneously solving the three-dimensional flow field within the GEU and the molecular diffusion of oxygen throughout the system. However, the flow in the GEU is horizontal, whereas oxygen transport by diffusion occurs mainly in the vertical direction. Under these assumptions, a simplified analytical model for the oxygen concentration in the liquid phase was developed (schematised in Figure 7).

Oxygen diffusion is described using Fick’s second law:

(1) $$\frac{\partial \;C_{O_{2}}}{\partial \;t}\;=\;D\frac{\delta^{2}\;C_{O_{2}}}{\delta \;z}$$

where $C_{O_{2}}$ is the dissolved oxygen concentration and *D* is the diffusion coefficient of oxygen in the liquid. The coordinate z denotes the vertical direction, corresponding to the dominant diffusion pathway. The diffusion time *t* results from the horizontal flow magnitude (and channel length). Consequently, oxygen transport can be modelled as one-dimensional diffusion in the vertical direction coupled with advection in the horizontal direction.

To simplify the equations, the flow profile is neglected, and diffusion of oxygen is assumed to take place in a static liquid. The residence time is determined by the horizontal position in the chip. The concentration of oxygen in the liquid entering the GEU is equal to:

(2) $$C_{O_{2}}\;\left(0\;<\;z\;<\;h\;,\;t\;=\;0\right)\;=\;C_{O_{2}}.0$$

Since the diffusion coefficient of oxygen is several orders of magnitude larger in the gas than in the liquid, we consider, as a first approximation, that diffusion of the oxygen in the gas phase is not limiting and that the concentration of the oxygen in the gas phase is homogeneous and constantly equal to 21%. The gas phase and the liquid phase are in equilibrium, and the concentration of oxygen in the liquid phase at the level of the interface is defined by the saturation concentration *C_sat_*. However, the exchange surface area between the two phases is limited by the porosity of the membrane, so that the overall concentration of oxygen at the interface is:

(3) $$C_{O_{2}}\;\left(h\;,\;t\right)\;=\;C_{O_{2,B}}\;=\;C_{sat}$$

In addition, the top of the GEU (*z* = 0) is impermeable and a no-flux boundary condition applies:

(4) $$\frac{\partial \;C_{O_{2}}}{\partial \;z}{|\;}_{z=0}\;=0$$

The solution to the differential equation (Equation (1)) with the initial and boundary conditions (Equations (2)–(4)) can be written explicitly [27].

(5) $$C_{O_{2}}\;\left(z\;,\;t\right)\;=\;C_{O_{2,0}}\;+\;\frac{4\left(C_{O_{2,B}}\;-\;C_{O_{2,0}}\right)}{\pi }\Sigma_{n=0}^{\infty }\;\frac{{\left(-1\right)}^{n}}{\left(2n\;+1\right)}.\exp\left(-\frac{D{\left(2n+1\right)}^{2}\pi^{2}t}{4L^{2}}\right)\;\cos\left(\frac{\left(2n+1\right)\pi z}{2L}\right)$$

The solution of this equation is plotted for different residence times in Figure 7B. For short durations (for example, durations smaller than the characteristic diffusion time), the exact description of the concentration of O_2_ at the membrane requires taking into account many terms in the series in Equation (5). In the present case, the solution is approximated numerically by limiting the series to 100 terms.

The concentration of oxygen in the liquid at the outlet of the GEU can be obtained by computing the average concentration over the height of a liquid column.

(6) $$C_{O_{2}.GEU\;outlet}\;=\;\bar{C_{O_{2}}\;\left(z\;,\;t\right)}\;=\frac{1}{h}\int_{0}^{h}C_{O_{2}}\;\left(z\;,\;Rt\;\right)\;dz$$

The calculations were processed with MATLAB R2024b software. The results of this equation are compared to the experimental results in Section 6.3.

## 5. Results

### 5.1. Deoxygenation Using Flask

**Figure 8:**
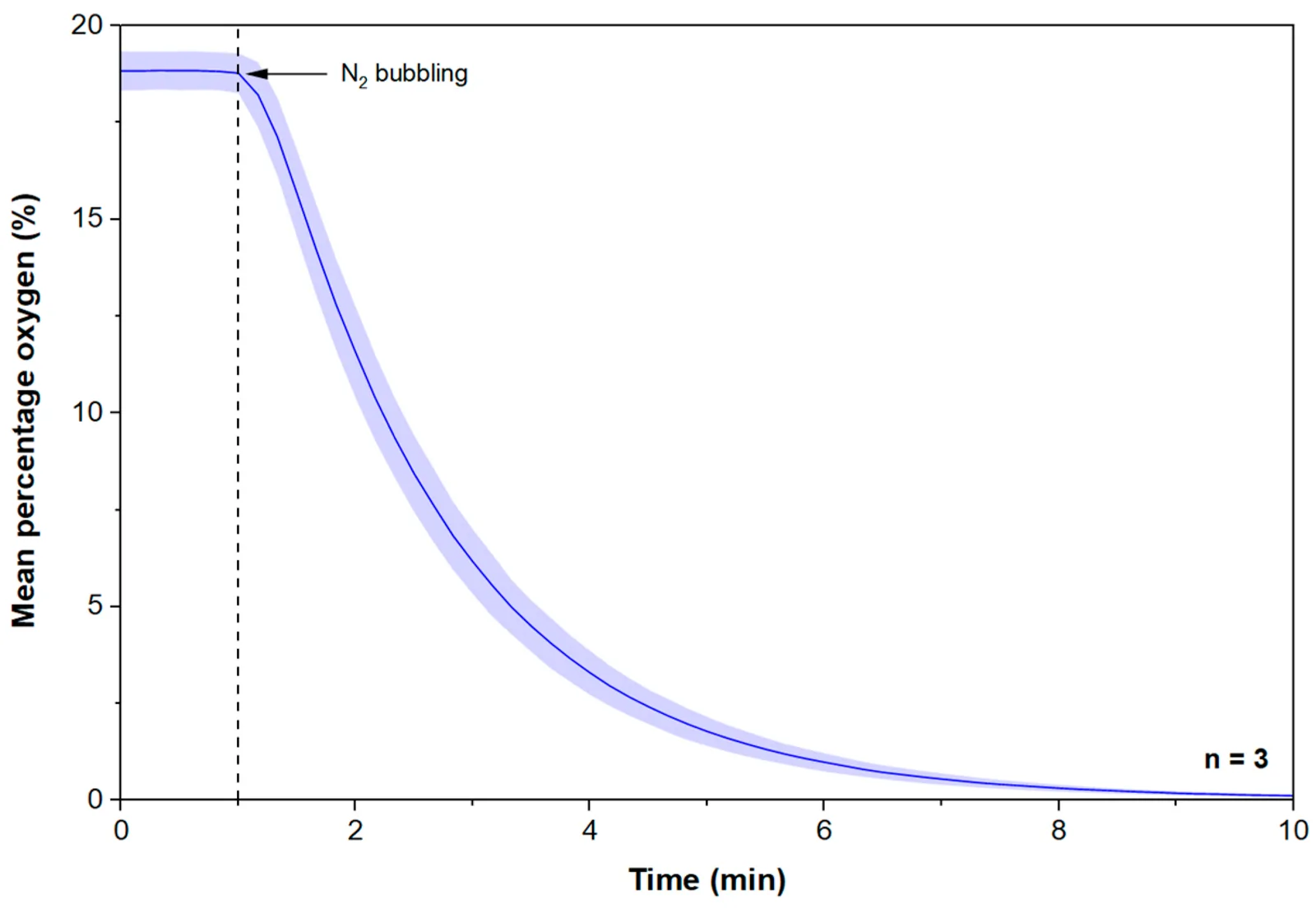
Mean change in oxygen level during deoxygenation inside flask (*n* = 3, shaded regions indicate standard deviation; data are presented as mean ± s.d.). From 0 min to 1 min (dashed line), there is no N_2_ bubbling. From 1 min to 10 min, 100 mbar N_2_ is bubbled in the deionised water.

In order to be able to carry out the experiments in Section 5.2, deoxygenated DI water had to be generated. This was achieved using the experimental setup in Figure 2. As shown in Figure 8, from 0 min to 1 min, there was no N_2_ bubbling and the mean oxygen level was 18.84%. N_2_ bubbling was started at 1 min, and immediately, the oxygen level started to decrease. At 3 min, the mean oxygen level was 6.16%, and at 6 min it was 0.98%. The N_2_ bubbling was stopped at 10 min, when the mean oxygen level reached 0.12%.

### 5.2. Reoxygenation Using GEU

**Figure 9:**
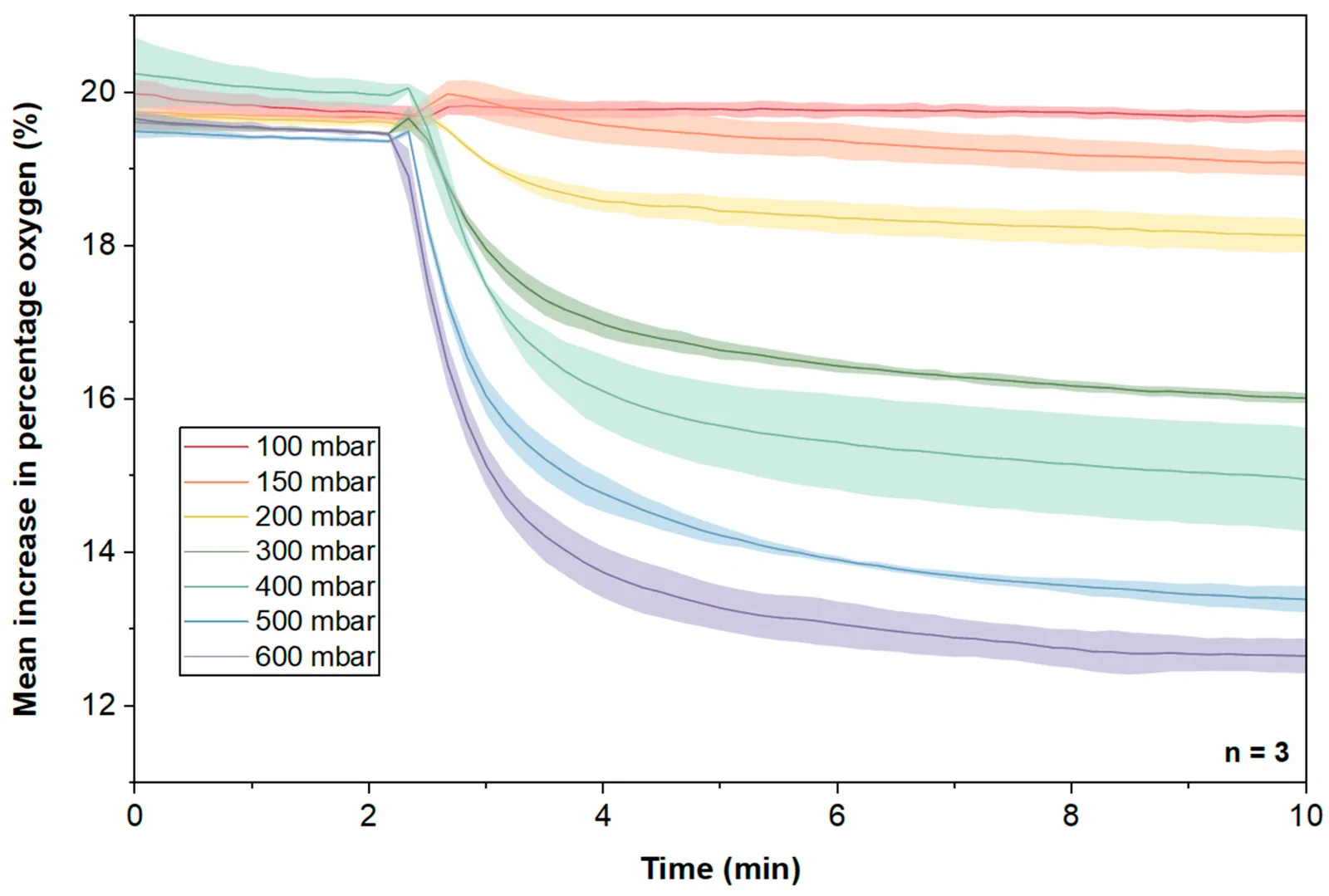
Mean increase in oxygen level during reoxygenation using GEU, without airflow in gas compartment (*n* = 3, shaded regions indicate standard deviation; data are presented as mean ± s.d.). From 0 min to 2 min, the liquid flow system was filled with air (internal control). From 2 min to 10 min, liquid pressures of 100, 150, 200, 300, 400, 500 and 600 mbar were applied.

In the first series of experiments (Figure 9), there was no airflow in the gas compartment (the inlet and outlet were left open). The mean increase in oxygen level was calculated by subtracting the mean oxygen level in the flask from the mean oxygen level in the FTOS at the outlet of the GEU. From 0 min to 2 min, the liquid flow system was filled with air (internal control). At 2 min, different pressures of N_2_ were applied to opening 2 of the flask (Figure 3), forcing deoxygenated DI water into the system at different flow rates. When 100 mbar was applied, the mean increase in oxygen level was 19.78% at 4 min and 19.69% at 10 min. At 10 min, the GEU was able to bring the oxygen level from a mean level of 0.09% (flask) to a mean level of 19.79% (outlet of GEU). When 600 mbar was applied, the mean increase in oxygen level was 13.74% at 4 min and 12.65% at 10 min. At 10 min, the GEU was able to bring the oxygen level from a mean value of 0.22% (flask) to a mean value of 12.88% (outlet of GEU). As the applied N_2_ pressure in the flask increased, the liquid flow rate in the GEU increased. This decreased the residence time and, subsequently, the reoxygenation efficiency.

**Figure 10:**
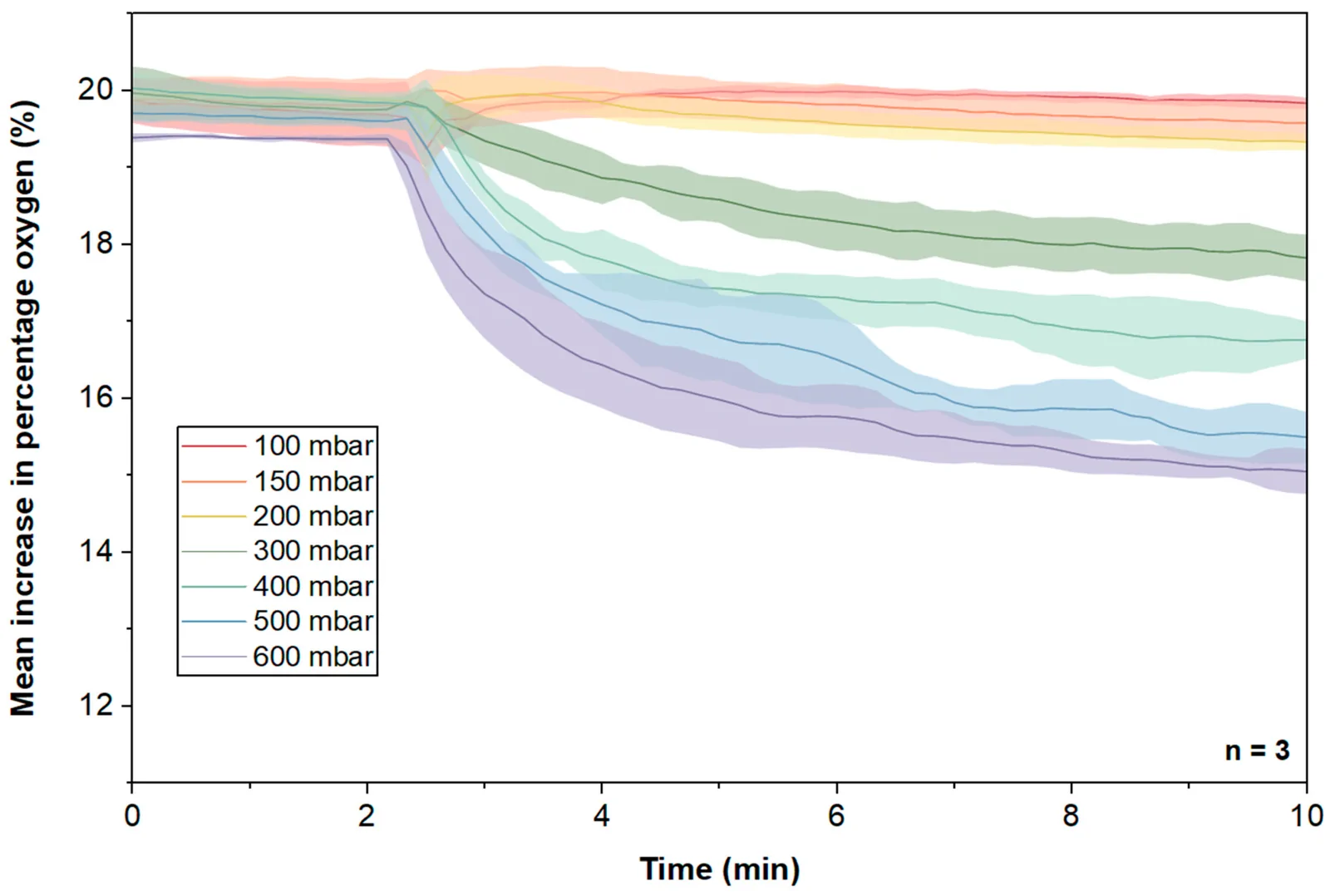
Mean increase in oxygen level during reoxygenation using GEU, with airflow in gas compartment (*n* = 3, shaded regions indicate standard deviation; data are presented as mean ± s.d.). From 0 min to 2 min, the liquid flow system was filled with air (internal control). From 2 min to 10 min, liquid pressures of 100, 150, 200, 300, 400, 500 and 600 mbar and gas pressures 50, 75, 100, 150, 200, 250 and 300 mbar, respectively, were applied.

**Figure 11:**
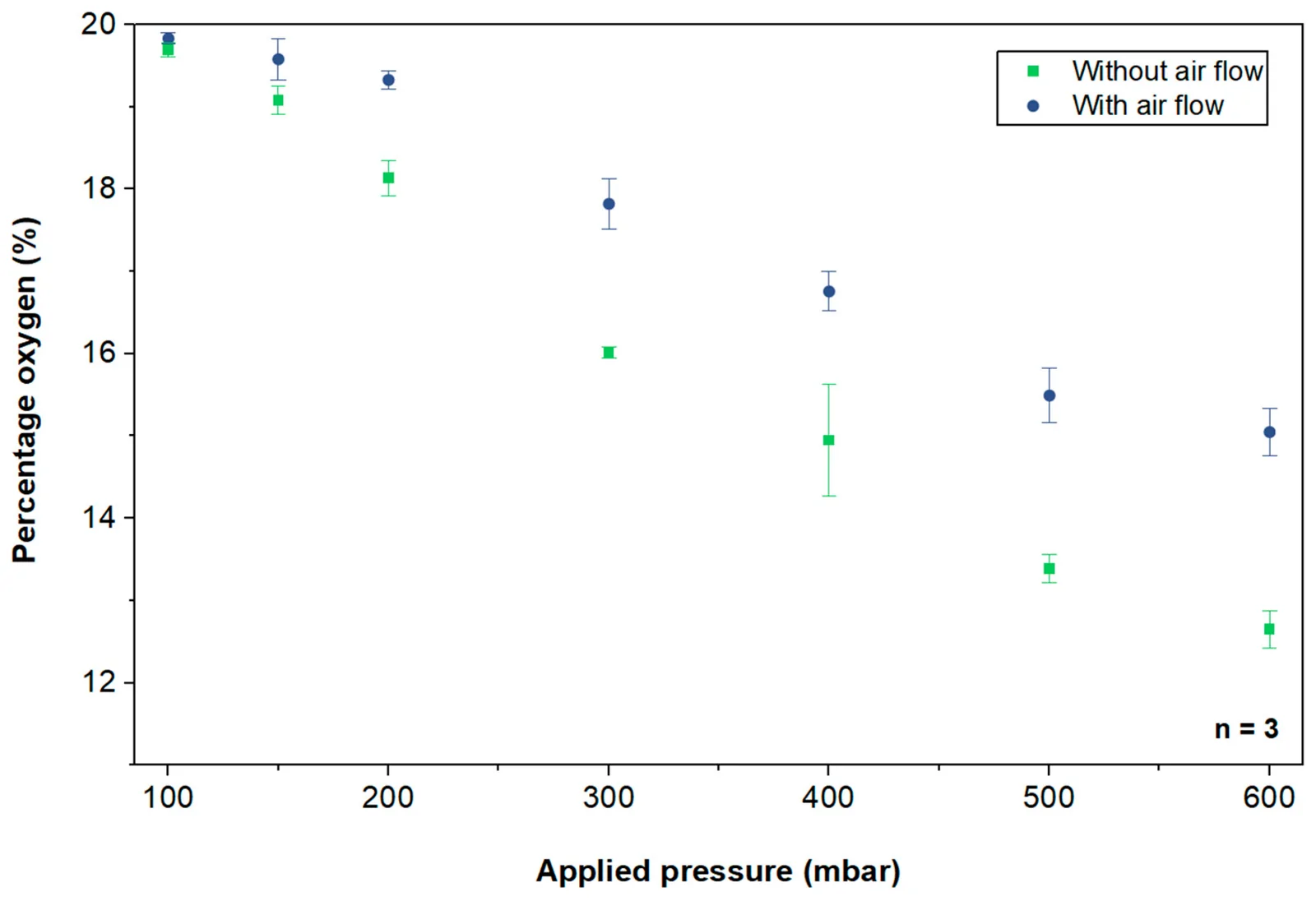
The mean increase in oxygen level at 10 min in the experiments without and with flow in the gas compartment of the GEU, for the different applied liquid pressures (*n* = 3; the error bars indicate standard deviation; data are presented as mean ± s.d.). The values correspond to the end values of Figure 9 and Figure 10.

In the second series of experiments (Figure 10), air was flowed through the gas compartment using a separate Fluigent pump. From 0 min to 2 min, the liquid flow system was filled with air (internal control). Figure 11 compares the mean increase in oxygen level at 10 min for the experiments without airflow (Figure 9) and with airflow (Figure 10) in the gas compartment of the GEU. Except for the experiments at 100 mbar, the mean oxygen level increase with airflow is significantly higher than the mean oxygen level increase without airflow. Hence, in general, airflow in the gas compartment enhances the reoxygenation efficiency of the GEU.

### 5.3. Deoxygenation Using GEU

**Figure 12:**
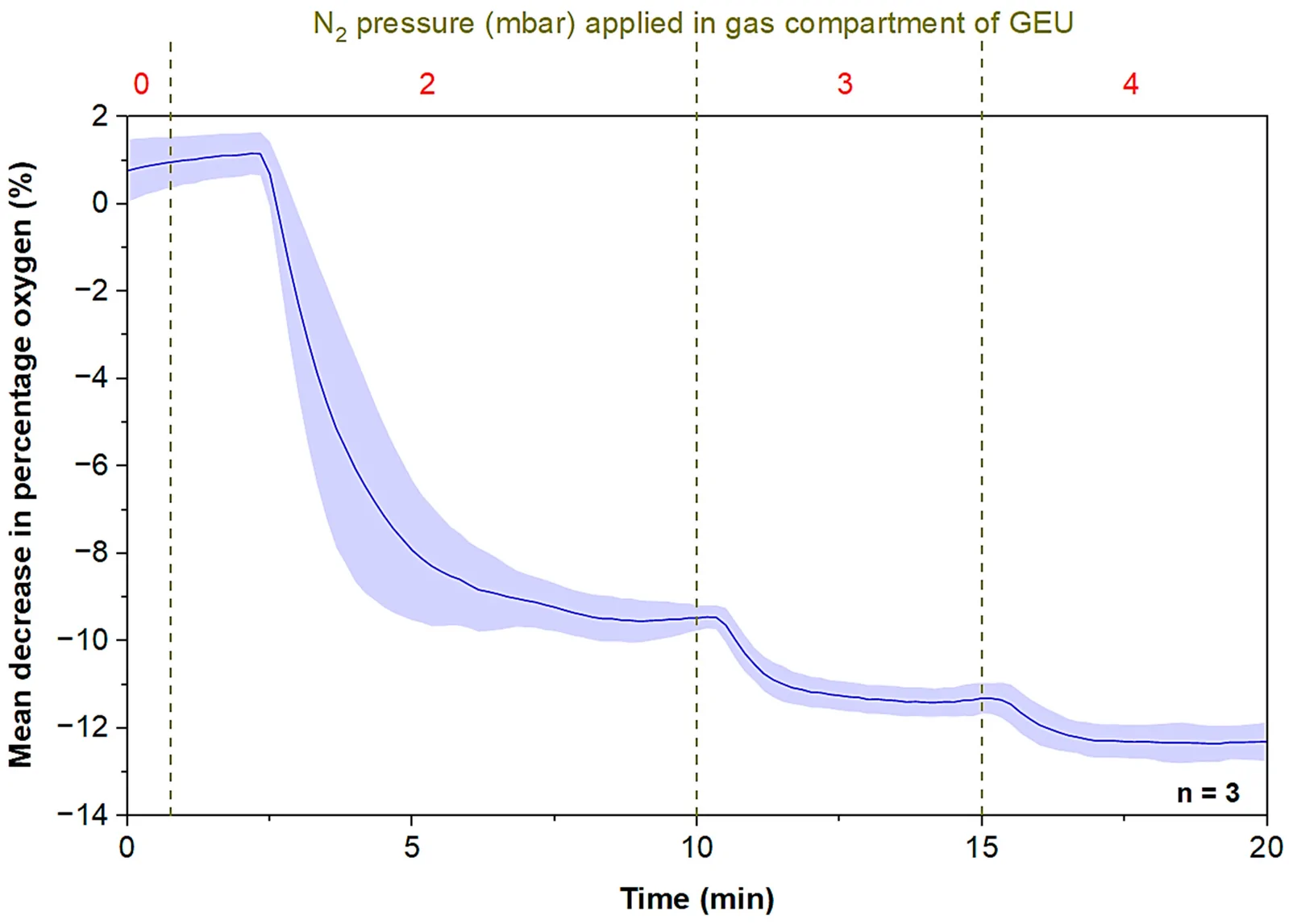
Mean decrease in percentage oxygen during deoxygenation using GEU (*n* = 3, shaded regions indicate standard deviation; data are presented as mean ± s.d.). A fixed liquid pressure of 200 mbar was applied throughout this experiment. From 0 min to 1 min, the gas pressure was 0 mbar. This was increased to 2, 3 and 4 mbar at 1, 10 and 15 min (dashed lines), respectively.

The mean change in oxygen level was calculated by subtracting the mean oxygen level in the flask from the mean oxygen level at the outlet of the GEU. The liquid flow was started at 0 min. As shown in Figure 12, from 0 min to 1 min, no N_2_ was flowed through the gas compartment of the GEU. From 1 min to 2 min, even though 2 mbar N_2_ was applied, the oxygen level kept increasing. This is because of the residual air inside the gas compartment. From 2 min to 10 min, there was a sharp decrease in the oxygen level. The decrease in oxygen level reached −9.48% at 10 min. From 10 min to 15 min, the N_2_ flow was increased to 3 mbar. The oxygen level decreased further. At 15 min, the decrease was −11.31%. From 15 min to 20 min, the N_2_ flow was increased to 4 mbar. The oxygen level decreased further. At 20 min, the decrease was −12.31%. Thus, varying the applied N_2_ pressure in the gas compartment of the GEU enabled a controlled deoxygenation of the DI water.

## 6. Discussion

### 6.1. Design of the Gas Exchange Unit

The conventional format for membrane contactors is the hollow-fibre configuration rather than the flat-sheet configuration [28]. The hollow-fibre configuration offers larger membrane surface area per unit volume and higher self-mechanical support [29]. However, in the current study, the flat-sheet membrane design was chosen because it is more suitable for cell culture applications. Membranes integrated in microfluidic cell culture systems are prone to biofouling from cell and protein mixtures [30], especially during recirculating perfusion. A potential strategy to minimise this is the incorporation of an upstream inline filter to remove larger particles and aggregates before the liquid enters the GEU, thereby reducing particulate accumulation within the device. The filter characteristics would need to be selected according to the application to minimise additional pressure drop while retaining the desired components of the culture medium. However, filtration alone may not prevent adsorption of proteins or other biological components onto the membrane. The modular design of the GEU allows the device to be disassembled, facilitating membrane cleaning or replacement when required. For prolonged operation, a combination of upstream filtration and periodic membrane maintenance could therefore be employed to minimise the impact of fouling.

### 6.2. Micropillars and Flow Profile Simulation

**Figure 13:**
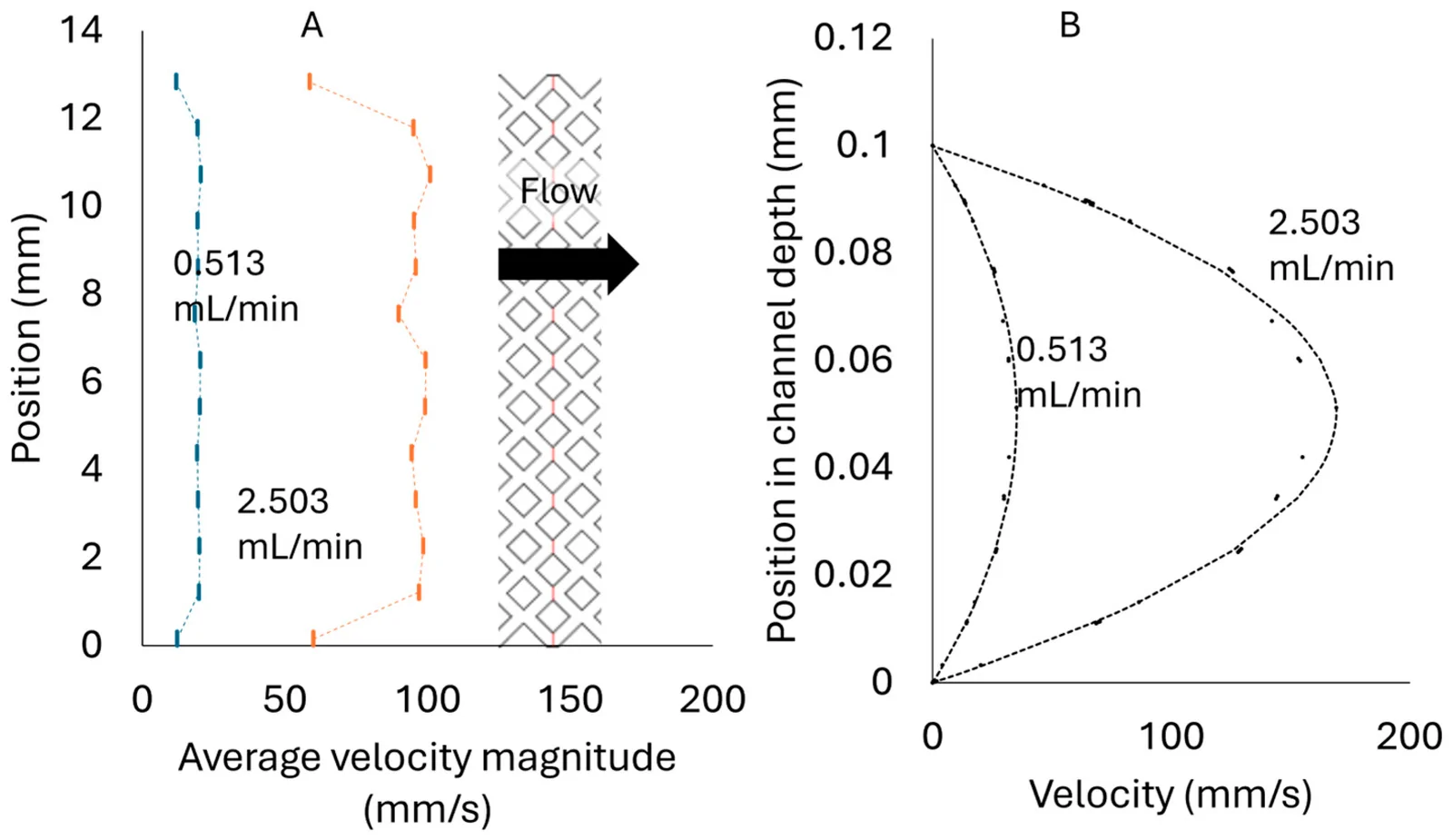
(**A**) Numerical simulation of average velocity in each channel across the device width in the micropillar region at half of the height (top view). The velocity is similar in all microchannels, demonstrating a uniform flow distribution. Only the two lateral channels show a slightly lower flow velocity. (**B**) The numerical simulation of the velocity as a function of the channel depth. A Poiseuille flow profile fit has been drawn (dashed line).

The presence of the micropillar array plays a crucial role in mechanically stabilising the membrane. In the absence of these supporting structures, the membrane would deform significantly under operating pressures [25], ultimately hindering or even preventing efficient oxygen exchange [25]. The flow exhibits a Poiseuille profile across the channel depth, while remaining relatively uniform along the device width, with slight deviations occurring close to the lateral boundaries, more pronounced at higher velocity. This can be observed from the numerical simulation of the flow in Figure 13A,B as well as in the literature [25].

The flow distributor of our design was inspired by a previous study carried out by our research group [25]. When the channel width is much wider than the feed zone, a proper flow distribution at the interface zone is crucial. In the absence of any flow guiding structures, the flow profile is severely warped [25]. This leads to a lower residence time of a liquid segment in the central part of the channel as compared to the zone near the sidewall. The residence time will determine the extraction efficiency, and the flow rate will be dictated by the residence time of the already further migrated central zone. The sidewall fraction will hence spend more time than necessary, and the overall effect will be a lower efficiency compared to when a perfect straight profile would be obtained. Due to the diamond-shaped structures, radial transport is strongly enhanced with respect to axial transport, resulting in a laterally straight flow profile. These structures can be very easily adapted to much larger ratios of initial and final channel width and can be implemented when conceiving designs for maximising the flow rates (and hence the channel width) [25].

In Figure 13A, it can be observed that in the diamond-shaped flow distributor, the outermost microchannels exhibit lower flow rates than the other channels. This behaviour arises from the topology of the distribution system: while the internal channels receive fluid from two neighbouring upstream pathways, each outer channel is supplied by only a single upstream branch. As a result, the edge microchannels carry a smaller fraction of the total flow, leading to lower average velocities compared with the channels in the central region of the distributor.

### 6.3. Comparison with Analytical Model

**Figure 14:**
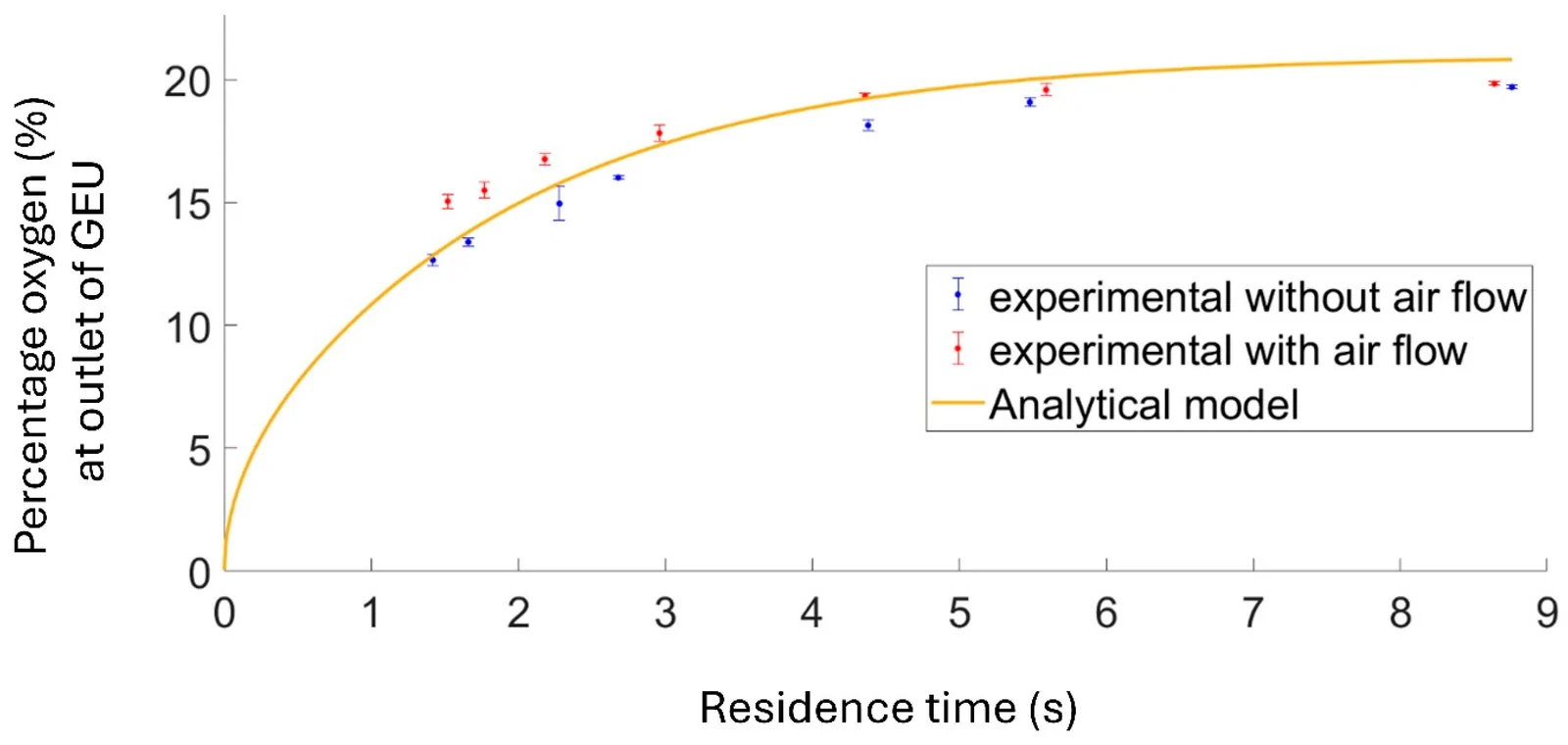
Comparison of experimental oxygenation levels obtained with and without airflow with the analytical model.

Figure 14 compares the experimental oxygenation levels obtained in the experiments (end values of the curves of Figure 9 and Figure 10) with the analytical model. The curve was calculated using Equation (6) (Section 4) with a saturation concentration of oxygen of 8.26 mg/L, considering that the liquid is perfectly deoxygenated before entering the GEU and using a value of 2.1 × 10^−9^ m^2^/s for the diffusion coefficient of oxygen in water. For low residence times, we observe a significant difference between the two datasets (with and without airflow). This indicates that, in the absence of airflow, the oxygen in the gas compartment is consumed and not sufficiently renewed through the two openings of the gas compartment, resulting in lower oxygenation. This effect is, however, limited because oxygen diffusion is fast and the oxygen saturation concentration in water is about 40 times lower than the concentration of oxygen in the air. In this region of the graph, the analytical model underestimates oxygenation in comparison with experimental results with airflow.

We hypothesise that it is because the model only describes diffusion, while in the experiments, the high flow rates induce mixing, mostly due to Taylor–Aris dispersion [31,32,33]. In the absence of airflow, these effects balance out, resulting in a good fit between the experimental data and the model. At low flow rates (high residence time), the model slightly overestimates oxygenation. This indicates that the membrane limits the transfer of oxygen, a phenomenon that is not addressed in the model, but this only seems to have a minor influence (Figure 14). In this regime, the two datasets result in similar oxygenation rates, indicating that oxygen has enough time to diffuse through the two openings of the gas compartment.

### 6.4. Controlling Oxygen Level

The GEU, in this study, demonstrated reoxygenation control between +12.65% (applied liquid pressure of 600 mbar) and +19.69% (applied liquid pressure of 100 mbar) without airflow in the gas compartment (Figure 9) and between +15.05% (applied pressure of 600 mbar) and +19.83% (applied pressure of 100 mbar) with airflow (Figure 10), when the starting oxygen level was <1%. According to the analytical model in Figure 14, further reoxygenation control below +12.65% (without airflow) and +15.05% (with airflow) can be achieved by increasing the applied liquid pressure and decreasing the residence time. The reoxygenation efficiency is higher with airflow in the gas compartment of the GEU (compared to without airflow), as shown in Figure 11.

Importantly, non-equilibrium operation is not necessarily advantageous when complete oxygenation is desired. However, for organ-on-a-chip applications, the target oxygen concentration may be substantially below the equilibrium concentration obtained with air. In this context, controlling the liquid residence time provides a simple means of limiting oxygen transfer and therefore generating an intermediate dissolved oxygen concentration. The GEU can consequently be operated either near equilibrium, when maximal oxygenation is required, or under non-equilibrium conditions to condition the liquid to a lower target oxygen level before it enters a downstream microfluidic device.

Engler et al. [11] reported an increase in reoxygenation efficiency with increasing liquid flow rate using a commercial hollow-fibre oxygenator. The difference from the trend observed in the present study is likely related primarily to the operating regime and the extent of gas–liquid equilibration. In the present GEU, increasing the liquid flow rate decreases the residence time and therefore limits the extent of oxygen transfer before the liquid exits the device.

The GEU also demonstrated deoxygenation control between −9.48% (applied N_2_ pressure of 2 mbar) and −12.31% (applied N_2_ pressure of 4 mbar), when the applied liquid pressure was 200 mbar, and the starting oxygen level was 18.08% (Figure 12). Deoxygenation control below −12.31% can be achieved by increasing the applied N_2_ pressure above 4 mbar. The deoxygenation efficiency can be further modulated by varying the applied liquid pressure and residence time.

For the reoxygenation experiments (Figure 9 and Figure 10), the liquid pressure was varied from 100 to 600 mbar, and the gas pressure from 50 to 300 mbar. The objective was to investigate how liquid residence time affected oxygen transfer. The purpose of the “Deoxygenation using GEU” experiment (Figure 12) was not to investigate the effect of liquid flow and residence time, but to demonstrate that the GEU can progressively control oxygen removal by changing the N_2_ supply. For these experiments, the liquid pressure was fixed at 200 mbar, and the gas pressure was varied from 0 to 4 mbar.

### 6.5. Integration of GEU with Microfluidic Cell Culture Devices

In this section, the potential integration of the GEU with a microfluidic cell culture device is discussed. It should be noted that, in the current study, the GEU was only characterised with deionised (DI) water and not with culture medium. This choice was made because the dissolved oxygen levels show significant variability between different culture media [34]. The analytical model in Section 4 can be easily adapted for different culture media. We expect the reoxygenation efficiency to be lower with culture medium, given its lower oxygen solubility, compared to water [35].

The GEU can be integrated in a single-pass perfusion system. In this configuration, the culture medium from a reservoir passes only once through the GEU and the microfluidic cell culture device before going directly to the waste. The GEU can be used to condition the culture medium with a specific oxygen level (reflecting in vivo oxygen levels) before it enters the microfluidic device. For example, the GEU can condition the culture medium with 5.4% oxygen for culturing hepatic cells.

The GEU can also be integrated in a recirculating perfusion system. In this configuration, the reservoir, the GEU and the microfluidic cell culture device form a closed loop. The culture medium is recirculated through the closed loop. During recirculation, as the cells in the microfluidic device continuously consume oxygen, continuous addition of oxygen into the culture medium is required. Controlled addition of oxygen can be achieved using the GEU. For example, the GEU can maintain a stable oxygen level of 5.4% for culturing hepatic cells in recirculating perfusion.

For optimal operation, the closed-loop system would also need to include a microcontroller similar to the one implemented by Jiang et al. [7]. The microcontroller could use the oxygen measurements from the oxygen meter to adjust the applied pressures of liquid, oxygen and nitrogen in the GEU in order to generate stable oxygen levels for specific cell types.

For integration with mammalian cell culture and organ-on-a-chip systems, the GEU would preferably be positioned inside the incubator or another temperature-controlled enclosure upstream of the microfluidic device. Maintaining the GEU, gas and liquid streams at the same temperature as the culture system is important because temperature influences oxygen solubility and oxygen mass transfer across the membrane. Consequently, changes in temperature can modify both the equilibrium dissolved oxygen concentration and the rate of oxygen transfer. Although the present study was performed at room temperature and did not systematically investigate the effect of temperature, temperature-controlled operation would be required for reproducible oxygen conditioning during cell culture applications.

For achieving a desired dissolved oxygen concentration, two complementary operating strategies can be considered. The GEU can be operated close to gas–liquid equilibrium using a gas mixture containing a defined oxygen concentration, or it can be operated under non-equilibrium conditions in which the extent of oxygen transfer is controlled by the liquid residence time. The latter approach allows the outlet oxygen concentration to be modulated while maintaining a fixed gas composition, such as air. In a single-pass configuration, a calibration curve relating liquid flow rate (or residence time) to outlet dissolved oxygen concentration could be established for defined gas pressure and temperature conditions, allowing the flow rate to be selected according to the desired oxygen concentration. In a recirculating configuration, the GEU could similarly be used to progressively adjust the oxygen concentration of the circulating medium. Gas mixing and residence-time control should therefore be regarded as complementary approaches, with the appropriate strategy depending on the desired oxygen concentration, system complexity and operating configuration.

### 6.6. GEU for Organ-on-a-Chip Applications

The GEU developed in this study potentially has organ-on-a-chip (OoC) applications. OoC systems aim to culture biological tissues in a controlled microenvironment, which reproduces in vivo conditions [36]. The oxygen level is a crucial microenvironment parameter. Ideally, OoC devices should reproduce physiological in vivo oxygen levels. For example, OoC devices for kidneys, liver and brain should reproduce oxygen levels of 9.5%, 5.4% and 4.4%, respectively [2]. Connecting a GEU before the OoC device and adjusting the liquid and gas flow rates would enable the desired oxygen level to be reproduced. Multi-organ-on-a-chip (Multi-OoC) combines several OoC devices [37]. The same culture medium flows through the different OoC devices, enabling cross-organ communication. One major issue with multi-OoC is that the different OoC devices for different organs/tissues require different oxygen levels. For multi-OoC with a plug-and-play configuration [38], this issue could be solved by connecting a GEU before each OoC device to condition the culture medium to the desired oxygen levels, as shown in Supplementary Figure S2.

## 7. Conclusions

In the current study, we developed a standalone PDMS-free Gas Exchange Unit (GEU) for controlled reoxygenation and deoxygenation of liquids for microfluidic applications. The device is fabricated using CNC-milled PMMA and incorporates a porous PTFE membrane for gas exchange. This GEU provides an alternative to PDMS-based oxygen-controlled strategies, avoiding limitations associated with PDMS, including small-molecule absorption, leaching and water evaporation.

Experimental characterisation demonstrated that the GEU efficiently modulates oxygen levels over a broad operating range. Reoxygenation efficiency decreased with increasing liquid flow rates due to reduced residence times. Airflow through the gas compartment enhanced reoxygenation by continuously replenishing oxygen at the membrane interface. On the other hand, controlled deoxygenation was achieved by flowing nitrogen through the gas compartment, with increasing nitrogen pressure producing progressively lower oxygen levels. Numerical flow simulations confirmed a uniform flow distribution throughout the device, and the analytical diffusion model successfully captured the overall oxygen transfer behaviour.

Unlike integrated oxygen-control approaches, the proposed GEU can be connected upstream of existing microfluidic platforms without requiring redesign or specialised fabrication processes. This flexibility makes the device particularly attractive for laboratories seeking to establish physiologically relevant oxygen environments in organ-on-a-chip and other microfluidic cell culture systems. The ability to both increase and decrease oxygen levels using the same platform provides a versatile tool for reproducing tissue-specific oxygen conditions.

Future work will focus on validating the GEU using mammalian cell cultures, evaluating long-term oxygen stability during single-pass and recirculating perfusion and integrating the device into organ-on-a-chip systems to demonstrate improved physiological relevance under controlled oxygen microenvironments.

## Data Availability

The data supporting the findings of this study have been deposited in Figshare under the DOI [10.6084/m9.figshare.33109073] and is accessible at [https://doi.org/10.6084/m9.figshare.33109073].

## Supplementary Materials

The following supporting information can be downloaded at: https://www.mdpi.com/article/10.3390/mi17091003/s1. Figure S1: Schematic of all the experimental setups for deoxygenation and reoxygenation; Figure S2: GEU connected before each OoC to condition culture medium with the desired oxygen level; Figure S3: Connecting GEU to the tubing; File S1: CAD drawing of GEU.

## Abbreviations

The following abbreviations are used in this manuscript:

CNC	Computer Numerical Control
DI	Deionised
Eq.	Equation
FTC	Flow-Through Cell
FTOS	Flow-Through Oxygen Sensor
GEU	Gas Exchange Unit
OoC	Organ-on-a-Chip
PC	Polycarbonate
PDMS	Polydimethylsiloxane
PMMA	Polymethyl methacrylate
PTFE	Polytetrafluoroethylene
