# Supplementary material for: Development of a Flat-Sheet Membrane Gas Exchange Unit for Oxygen Control in Microfluidic Systems
Source: Micromachines (Basel). 2026 Aug 25;17(9):1003. doi: 10.3390/mi17091003 (PMC13609301; doi:10.3390/mi17091003)
Supplement: Supplementary file 1 [file micromachines-17-01003-s001.zip › micromachines-4497434-Supplementary Figures S1-S3.pdf]

Supplementary materials

Development of a flat-sheet membrane Gas Exchange Unit for oxygen control in microfluidic systems

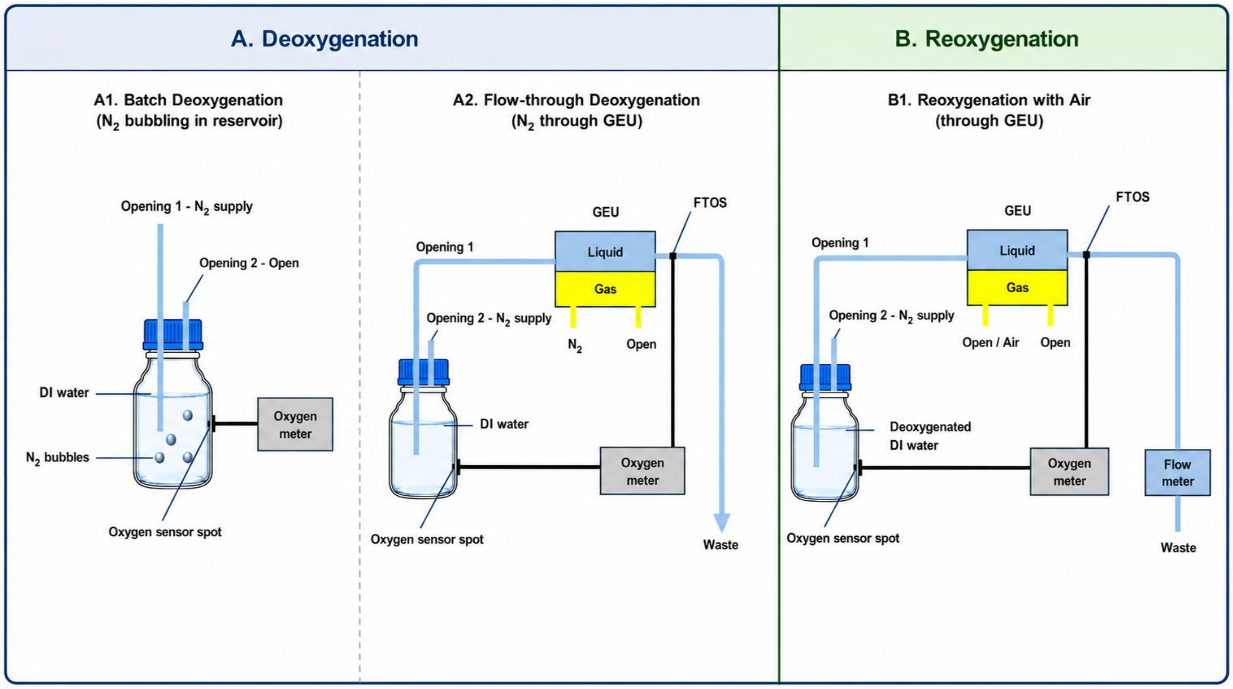

Supplementary Figure S1: Schematic of all the experimental setups for deoxygenation and reoxygenation.

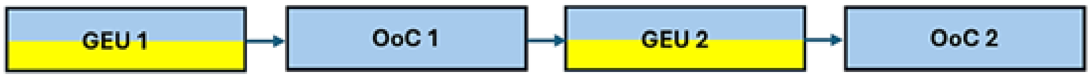

Supplementary Figure S2: GEU connected before each OoC to condition culture medium with the desired oxygen level.

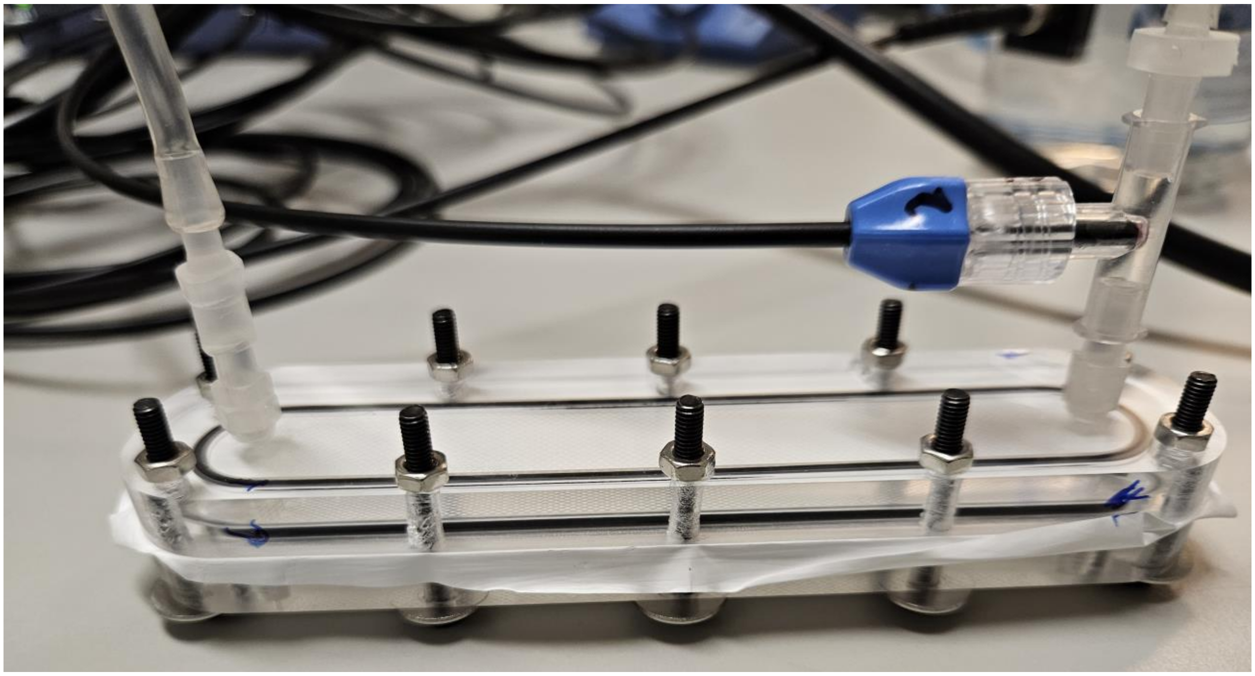

Supplementary Figure S3: Connecting GEU to the tubing - The liquid inlet tube coming from the flask is connected to a 3/32" Barbed to Female Luer Lock Adapter (P-858 / IDEX / USA). This then connects to a Male Slip Luer (10-32 Male UNF) (CIMLSTA-1032M-PC / ISM / USA) which is then screwed into the 10-32 threaded hole inlet of the GEU. The same connections are used to connect the gas line to the inlet of the gas compartment. At the outlet of the liquid compartment of the GEU, a Male Slip Luer (10-32 Male UNF) is screwed into the 10-32 threaded hole. This then connects to the Flow Through Oxygen Sensor (FTC-PSt3-YOP-4mm / PreSens / Germany).
